# The Role of KRAS Mutation in Colorectal Cancer-Associated Thrombosis

**DOI:** 10.3390/ijms242316930

**Published:** 2023-11-29

**Authors:** Radu Andrei Emilescu, Mariana Jinga, Horia Teodor Cotan, Ana Maria Popa, Cristina Maria Orlov-Slavu, Mihaela Cristina Olaru, Cristian Ion Iaciu, Andreea Ioana Parosanu, Mihaela Moscalu, Cornelia Nitipir

**Affiliations:** 1 Faculty of Medicine, Carol Davila University of Medicine and Pharmacy, 8 Sanitary Heroes Boulevard, 050474 Bucharest, Romania; raduemilescu66@gmail.com (R.A.E.);; 2 Preventive Medicine and Interdisciplinarity Department, Faculty of Medicine, Grigore T. Popa University of Medicine and Pharmacy, 16 University Street, 700115 Iasi, Romania

**Keywords:** molecular subtypes, arterial and venous thrombosis, KRAS, colorectal cancer

## Abstract

Venous thromboembolic events (VTE) are common in patients with colorectal cancer (CRC) and represent a significant contributor to morbidity and mortality. Risk stratification is paramount in deciding the initiation of thromboprophylaxis and is calculated using scores that include tumor location, laboratory values, patient clinical characteristics, and tumor burden. Commonly used risk scores do not include the presence of molecular aberrations as a variable. This retrospective study aims to confirm the link between KRAS-activating mutations and the development of VTE in CRC. A total of 166 patients were included in this study. They were split into two cohorts based on KRAS mutational status. We evaluated the frequency and mean time to VTE development stratified by the presence of KRAS mutations. Patients with mutant KRAS had an odds ratio (OR) of 2.758 for VTE compared to KRAS wild-type patients, with an increased risk of thrombosis being maintained in KRAS mutant patients even after adjusting for other known VTE risk factors. Taking into account the results of this study, KRAS mutation represents an independent risk factor for VTE.

## 1. Introduction

Venous thromboembolic events (VTE) are common in cancer patients and represent a major cause of morbidity and mortality. While the correlation between cancer and thrombosis was first described by Trousseau in 1867 [1], there was no consensus regarding the etiology connecting the two. The first modern cohort study published on the subject showed a 9% incidence rate of occult malignancy in patients diagnosed with thromboembolic disease [2].

Multiple modern studies have confirmed this correlation, proving that cancer patients had a much higher rate in VTE compared to the general population, with 18 to 29% of all diagnosed VTE being associated with an active malignancy [3,4,5,6,7]. Cancer is an important risk factor for the development of VTE, the Olmsted County population study reported a four times higher risk of VTE for patients with active cancer compared to the general population [6]. A more recent population-based case-control study on the Dutch population (MEGA study) shows a sevenfold increased VTE risk in patients with active cancer [8].

The cumulative incidence of VTE in cancer patients varies widely between 1% and 8% due to the heterogeneity in cancer type, cancer stage, patient population, duration of follow-up and VTE detection method [9]. A meta-analysis by Horsted et al. illustrates VTE incidence rate stratified by the baseline risk of venous thrombosis. Average-risk patients, defined as patients representative of all patients with cancer, had an incidence rate of 13 cases per 1000 person-years. For high-risk patients, defined as patients with advanced or metastatic cancer and patients treated with antineoplastic agents that increase thromboembolic risk such as anti-VEGFR (vascular endothelial growth factor receptor) monoclonal antibodies, the incidence rate was 68 cases per 1000 person-years [10].

**Figure 1:**
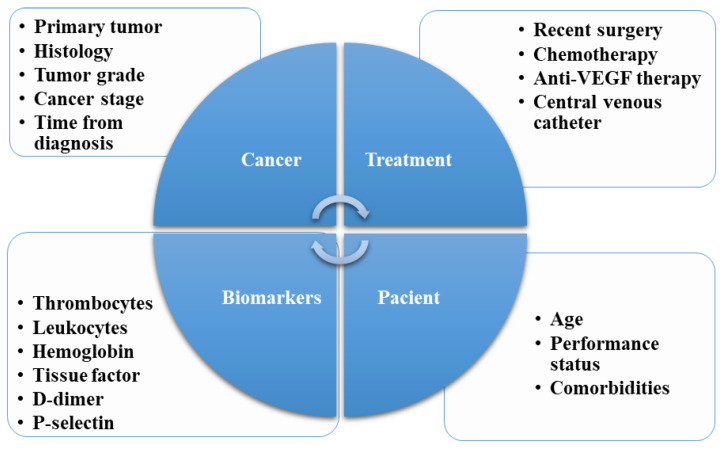
Cancer patients risk factors for developing VTE.

Stratifying the risk of thrombosis is a difficult task due to the large amount of possible risk factors that must be taken into account. Risk factors can be classified by how they relate to the patient clinical characteristics, treatment, laboratory values, and cancer characteristics (Figure 1).

Cancer is a heterogeneous disease, the main cancer-related risk factors for thrombosis are cancer subtype, histology, tumor grade, cancer stage, and time from cancer diagnosis.

Numerous studies have been published on the relation between cancer type and the subsequent risk of VTE, with pancreas, lung, brain, ovarian, stomach, and bone cancer being associated with the highest risks. Low risk was usually associated with breast, prostate, and thyroid cancer [10,11,12,13,14].

It is apparent that more aggressive cancer types, as evidenced by short survival times and early metastatic dissemination, are correlated with a higher incidence of venous thrombosis [15]. The correlation between the aggressiveness of cancer and thrombogenic risk is also observed when taking the cancer stage into account, with a higher stage being associated with a higher risk of thrombosis. In a Danish populational study that included over 40,000 cancer patients, VTE risk strongly correlated with cancer stage, with an HR of 2.9, 2.9, 7.5, and 17.1 for stages I, II, III, and IV respectively [16].

Different histological subtypes have also been associated with varied VTE risks, although these correlations are not as clear. For example, certain subtypes of ovarian, lung, and pancreas cancer show a higher incidence of VTE (lung adenocarcinoma, high-grade ovarian cancer, mucin-producing pancreatic adenocarcinoma) [9,17], while histology was not found to be a predictive risk factor for colon or breast cancer [18,19]. Ahlbrecht et al. showed that patients with high-grade cancer (G3–G4) had twice the risk of developing VTE compared to patients with low-grade cancer (G1–G2), irrespective of cancer type [20].

A retrospective study by Metcalf RL et al., showed a comparatively high risk of VTE in CRC, with a cumulative incidence as high as 8.9% [21]. Colorectal surgery did not appear to increase VTE risk in CRC patients if standard postoperative thromboprophylaxis regimens were followed [22].

Several risk stratification models that utilize clinical and laboratory characteristics exist [23,24], the most well-validated being the Khorana score [25]. The performance of the Khorana score in CRC is still not well established, although there are data that show a correlation between patient death and an increased Khorana score [21,26].

The introduction of routine molecular testing of tumor and blood samples has resulted in a dimension of cancer classification beyond histological type and location. Given the immense role of tumor genetics in the modern approach to cancer management the question arises: “Do tumoral mutations play a role in thrombotic risk?”.

Routine testing for some tumoral mutations (such as RAS, more particularly KRAS [Kirsten rat sarcoma viral oncogene]) has become standard practice due to their predictive value regarding response to certain treatments [27].

The KRAS gene is located on chromosome 12 and is a part of the RAS family of genes associated with human tumor development. It codes for a membrane-associated small GTPase that plays a crucial role in cell proliferation, differentiation, and survival by acting as an on/off molecular switch, alternating between active and inactive signal-transducing conformations. KRAS mutations lead to stabilization of the protein in the “on” state, with subsequent amplification of downstream signaling pathways leading to increased cell proliferation, a contributor to tumorigenesis. Exon 2 of the KRAS gene has a high mutation rate, the most common point mutations being glycine at position 12 (G12) and position 13 (G13) [27,28]. KRAS mutation is a relatively common activator mutation, with approximately 42–52% [27,29] of CRC patients harboring a form of KRAS mutation.

This study aims to establish whether a valid association between VTE risk and KRAS-activating mutations in colorectal cancer exists, a step necessary towards the development of more up-to-date thromboprophylaxis protocols.

## 2. Results

### 2.1. Patient Characteristics

**Figure 2:**
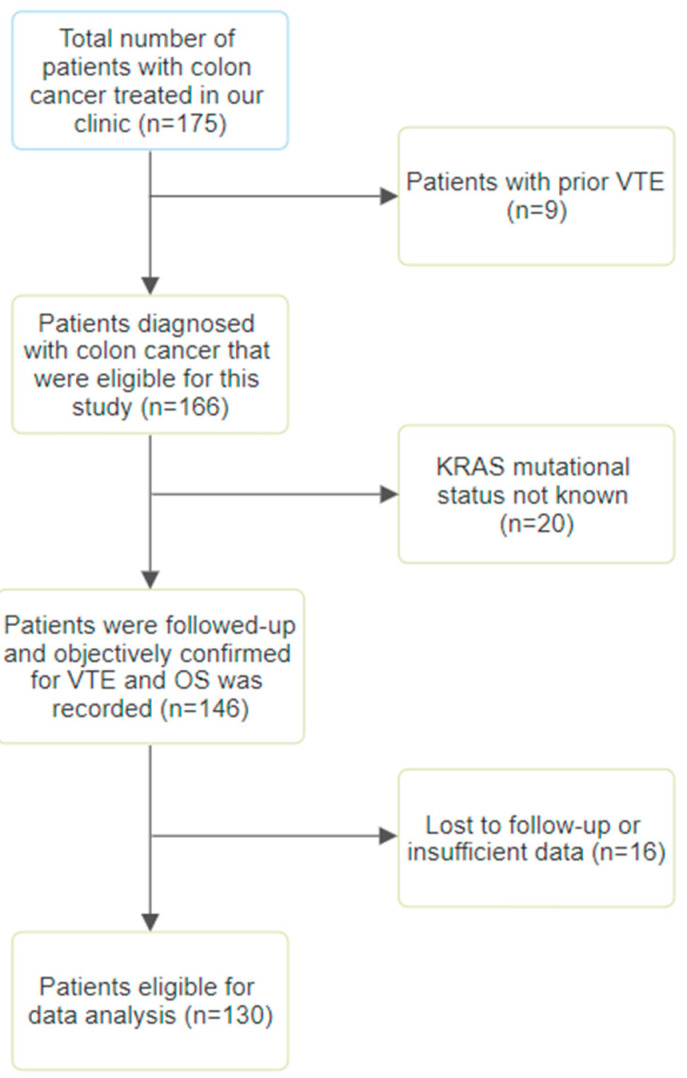
Study flow diagram.

This retrospective study identified 175 patients with colorectal cancer. After the exclusion of 45 patients due to a lack of KRAS mutational status, prior VTE, or insufficient clinical data, a total of 130 patients were eligible for statistical analysis (Figure 2). Demographically, 60 (46.2%) were male and 70 (53.8%) female, with a mean age of 67.3 years (SD ± 10.7). A majority of patients (*n* = 70, 53.8%) were TNM stage IV, 53 (40.8%) were stage III, and only 7 (5.4%) were stage II. Khorana score was calculated for all 130 patients, 109 (83.8%) had a Khorana score of <2 which was considered low risk for cancer-associated thrombosis (CAT) and 21 patients (16.2%) had a Khorana score of ≥2 which was considered as high risk for CAT. A total of 45 patients (34.6%) had developed a VTE, of which 30 (23.07%) were peripheral deep vein thromboses and 15 (11.53%) were pulmonary embolisms.

A total of 87 (66.9%) patients were classified as KRAS wild-type and 43 (33.1%) had a KRAS-activating mutation. KRAS mutation was almost equally distributed among male and female patients. Treatment that included anti-VEGF agents was administered to thirty-two (24.6%) patients, of which twenty-seven had a KRAS-activating mutation and only five were classified as KRAS wild type.

Patients’ descriptive statistical information stratified by KRAS mutational status is represented in Table 1.

### 2.2. Survival and VTE

**Figure 3:**
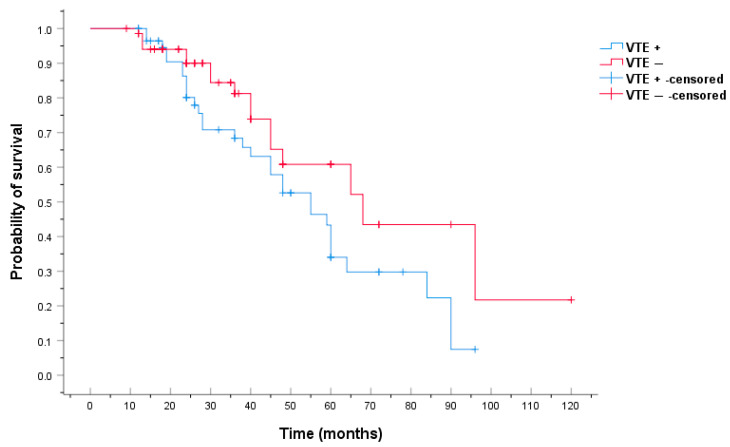
Comparative analysis of Kaplan–Meier curves between VTE-positive and VTE-negative patients for overall survival (OS).

Median overall survival time was 55 months (SD ± 7.662, 95% CI 39.982–70.018) for patients that developed a VTE and 68 months (SD ± 14.115, 95% CI 40.334–95.666) for patients with no VTE (Figure 3). The 3-year OS rate was 65.2% for patients that developed VTE and 82.6% for patients that did not develop VTE (Table 2).

We performed a univariate Cox regression which showed that KRAS mutation did not represent a significant risk factor for OS (HR = 1.721, *p* = 0.236). Data from the multivariate Cox regression analysis showed that OS was significantly influenced by ECOG performance status (HR = 1.329, *p* = 0.01), clinical stage (HR = 1.688, *p* = 0.09), age (HR = 1.388, *p* = 0.045), and Khorana score (HR = 3.131, *p* = 0.02). This data is showcased in Table 3.

### 2.3. The Association between KRAS Mutational Status and VTE Risk

The median time until VTE development for the identified cohort was 48.2 months (SD ± 17.039, 95% CI 14.603–81.397). For KRAS mutant patients the median time until VTE development was 12 months (SD ± 3.513, 95% CI 5.115–18.885). The median time for KRAS wild-type patients was not reached.

In a univariate logistic regression, KRAS mutational status was correlated with an increased risk of thrombosis, with an odds ratio (OR) of 2.758 (95% CI: 1.552–4.903, *p* = 0.001) for the general outcome of VTE, while the OR for DVT was 3.125 (95% CI: 2.537–5.039, *p* = 0.002) and for PE was 1.752 (95% CI: 1.239–3.756, *p* = 0.045).

Table 4 shows the results of logistic regression adjusted for known risk factors of VTE. We found that the correlation between KRAS and DVT/PE was statistically significant even after adjustment for Khorana score, Bevacizumab use, and clinical stage.

At 12 months the probability for VTE development was 20.2% and 51.4% in patients with KRAS wild-type and mutant KRAS, respectively (log-rank *p* = 0.001) (Table 5).

We also performed a Cox regression that included age (>65 years vs. <65 years), sex (M vs. F), tumor stage (II–III vs. IV), performance status (ECOG 0–1 vs. 2–3), Khorana score (<2 vs. ≥2), and treatment (regimens with anti-VEGF agent vs. other) to identify factors that contribute to the development of VTE. Male sex (HR = 1.698, 95% CI = 0.923–3.178, *p* = 0.036), poor performance status (HR = 2.030; 95% CI = 1.152–3.579, *p* = 0.014), KRAS-activating mutation (HR = 2.538, 95% CI 1.317–4.890, *p* = 0.005), and a ≥2 Khorana score (HR = 1.466; 95% CI 0.976–3.288, *p* = 0.015) were associated with an increased risk of VTE development. Age, treatment with bevacizumab, and tumor stage were not associated with a greater risk of developing VTE (Table 6).

**Figure 4:**
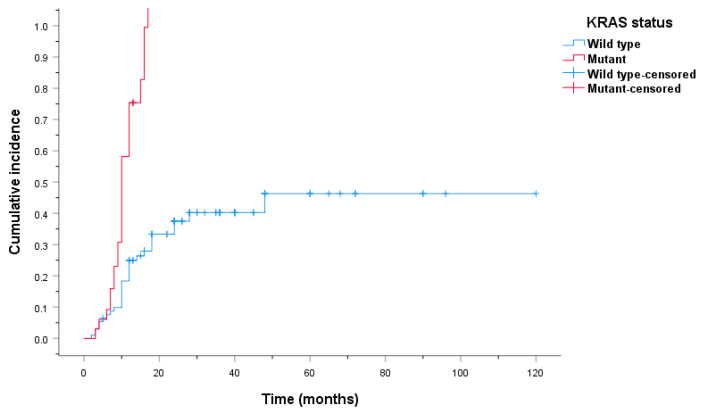
Cumulative incidence of VTE among CRC patients with and without KRAS mutations.

We have also established the cumulative incidence of VTE among CRC patients with and without KRAS mutations. Patients with KRAS mutations exhibited an increased rate of VTE, with a median time until development of 12 months, which is in stark contrast to KRAS wt patients, which have not reached the median (Figure 4).

The rate of VTE development at 9 months was 26.5% for KRAS mutant patients and 8.4% for KRAS wt patients. The rate at which VTE develops increased rapidly for KRAS mutant patients, with 66.4% of patients exhibiting VTE at 24 months compared to KRAS wt patients, of which only 22.1% had developed VTE at 24 months.

## 3. Discussion

CRC is frequently associated with rectal bleeding and anemia [30] and deciding to initiate thromboprophylaxis is problematic. Identifying CRC patients with an additional risk of developing thrombosis can facilitate management and have a beneficial impact on patient care. Several risk stratification models that utilize clinical and laboratory characteristics exist [23,24], the most well-validated being the Khorana score [25]. The performance of many of these models, including the Khorana score, is not well established in CRC, although there are data that show a correlation between patient death and an increased Khorana score [31]. In our study, patients with an increased Khorana score (≥2) had a 3-fold increase in the risk of death and a 1.46 times increase in the risk of developing thrombosis, consistent with the literature [31].

A study by Andes et al. that included 172 patients with metastatic CRC reported an incidence of 32.3% for VTE in patients with KRAS mutations vs. 17.8% for patients with wild-type KRAS [32]. Different papers that analyzed KRAS mutation in other cancers such as non-small lung cancer did not reach a clear conclusion [33]. Our study reports a 2.75-fold increase in the incidence of VTE in patients with mutant KRAS compared to patients with wild-type KRAS. KRAS mutation was an independent predictor of VTE even after adjustment for other common thrombosis risk factors such as clinical stage, Khorana score, and anti-VEGF agent use. Our statistical analysis showed that KRAS mutation had the biggest impact on thrombosis risk in the subgroup with a low Khorana score, suggesting a possible predictive value when other thrombosis risk factors are not present. Compared to Andes et al. [32], we used different Khorana score cutoffs for high (≥2) and low (<2) thrombosis risk. We believe that using a high-risk and low-risk classification for the Khorana score can facilitate patient selection and monitoring. Although different, the classification used in this study remained statistically relevant.

We hypothesize that the link between KRAS mutation and the increased risk of thrombosis is related to the underlying mechanisms of cancer-associated thrombosis. A recent study by Nasser et al. [34] divided hypercoagulability into two categories: Type I occurs when there is an imbalance between endogenous heparin production and its degradation by an enzyme called heparanase and Type II includes hypercoagulability caused by factors such as stasis, treatment, poor performance status etc. The role of heparanase in CAT has been verified by a series of studies, most of which have concluded that heparanase expression is associated with oncogene expression, including RAS mutations [35,36,37,38]. Another possible link between the risk of thrombosis and KRAS mutation is suspected based on data suggesting that tissue factor (TF), a main contributor to CAT, is over-expressed in KRAS mutant malignant cells [39]. An interrelation between TF and carcinogenesis has also been hypothesized. TF is involved in cancer-related processes such as tumor growth, angiogenesis, and metastasis, suggesting that variables commonly associated with an increased risk of thrombosis such as a higher clinical stage or increased cancer aggressiveness could be causing CAT via elevated TF expression [40]. In addition to KRAS mutation, other aberrations in molecules such as ALK, ROS 1, EGFR, and PTEN are also associated with an increased risk of thrombosis due to increased TF expression [41].

Several other studies have expanded upon the subject of oncogene-associated thrombosis. A meta-analysis by Liu et al. reported that NSCLC (non-small lung cancers) with ALK (anaplastic lymphoma kinase) or ROS1 (ROS proto-oncogene 1 receptor tyrosine kinase) were more likely to develop thrombosis than patients without these molecular aberrations. Furthermore, thrombosis was also associated with an inferior response to TKI (tyrosine kinase inhibitors) therapy and an inferior prognosis [42]. The mechanism behind the more frequent development of thrombosis in ALK/ROS1-mutated NSCLC is unknown, although several hypotheses have been made. Firstly, ALK/ROS1-mutated tumors are mucin abundant, and mucin generates signals that result in thrombocyte aggregation and thrombosis. Secondly, the co-occurrence of prothrombotic and oncogene mutations could explain the higher rate among ALK/ROS1-mutated NSCLC. Another possible mechanism is the cross-talk of ALK/ROS1 downstream signaling with procoagulant factors such as TF.

Another study by Dou et al., explored the impact of EGFR (epidermal growth factor receptor) and KRAS mutation in NSCLC. The study reported that EGFR mutations were associated with a decreased risk of VTE while KRAS mutations were associated with an increased risk of VTE, although this association was not statistically significant [33].

A study by Dunbar et al., analyzed the impact of various mutations irrespective of cancer histology on the risk of VTE development. Mutations in STK11, CDKN2B, KEAP1, CTNNB1, MET, and KRAS were associated with a significantly increased risk of cancer-associated thrombosis. Several mutations such as IDH1 and SETD2 had a decreased risk of VTE development [43].

Taking into account the increased risk of bleeding and VTE in cancer patients, a better selection of patients eligible for thromboprophylaxis is necessary. Classical biomarkers such as D-dimer, fibrinogen, and coagulation factors levels, as well as thrombocyte count, can be complemented by more precise biomarkers such as TF-bearing microparticles, soluble P-selectin levels, and even the presence of tumor molecular aberrations.

## 4. Materials and Methods

### 4.1. Patient Selection and Data Collection

Medical records were reviewed for all CRC patients whose treatment and follow-up took place at the Elias Emergency University Hospital, Bucharest, Romania between January 2012 and October 2022. The inclusion criteria represented CRC diagnosis confirmed by histopathology and tumor KRAS mutation testing.

We performed a data review looking to extract pre-chemotherapy blood count, body mass index, patient treatment, occurrence of VTE, demographic data, pretreatment performance status, and CRC stage as well as date of diagnosis. VTE were defined as the presence of deep vein thrombosis (DVT) or pulmonary embolism (PE), at any time after the diagnosis of CRC.

The Khorana score was used for risk stratification. We have opted for the Khorana score that was evaluated in the AVERT and CASSINI trials with a cutoff value of 2 (low risk of VTE < 2 and high risk of VTE ≥ 2). TNM classification 8th edition criteria were used for tumor staging. Eastern Cooperative Oncology Group (ECOG) classification was used to establish performance status at diagnosis.

Exclusion criteria consisted of active treatment with anticoagulation agents at the time of diagnosis, presence of VTE before cancer diagnosis, lack of KRAS mutational status testing as well as lack of body mass index and pre-chemotherapy laboratory results. We mention that port-a-cath use was not taken into consideration as a risk factor.

### 4.2. Laboratory and Imaging Methods

DNA was extracted from the formalin-fixed, paraffin-embedded (FFPE) tissue sample as well as from blood samples using two kits for this purpose: the QIAmp DNA FFPE tissue kit and the QIAmp DSP DNA Mini kit (Qiagen, Bucharest, Romania). After DNA extraction, a targeted resequencing assay was performed using the Ion AmpliSeq NGS Panel from Thermo Fisher Scientific (Waltham, MA, USA). This procedure involves amplifying specific regions of the genome (in this case, exons 2, 3, and 4 of the KRAS gene) using custom-designed primer sets. We considered the patient to have mutant KRAS status if any of these mutations were identified. The absence of any of the screened mutations was considered as KRAS wild-type status. We mention that we only considered somatic KRAS mutations as relevant for this study.

DVT was identified using Doppler echography, while contrast computed tomography (CT) was used for PE. During patient follow-up, any suspicion was followed by screening in the case of both DVT and PE.

### 4.3. Laboratory Methods Procedures

The QIAamp DSP DNA FFPE Tissue Kit uses special lysis conditions to release DNA from tissue sections and to overcome inhibitory effects caused by formalin cross-linking of nucleic acids with the purpose of DNA purification from formalin-fixed paraffin-embedded tissues. The procedure consists of 6 steps: remove paraffin, lyse, heat, bind, wash, and elute. Paraffin is dissolved in xylene and removed. The sample is lysed under denaturing conditions, with a short proteinase K digestion. Incubation at 90 °C reverses formalin cross-linking. DNA binds to the membrane and contaminants are washed away. DNA is eluted in Buffer ATE (low-EDTA [ethylenediaminetetraacetic acid] elution buffer optimized for long-term storage of DNA) and is immediately ready for use in amplification reactions or for storage at −20 °C. The simple QIAamp DSP DNA FFPE Tissue Kit procedure is highly suited for the simultaneous processing of multiple samples.

The QIAamp DSP DNA Mini Kit procedure, using QIAamp Mini spin columns, has four simple steps: lyse, bind, wash twice, and elute. The QIAamp DSP DNA Mini Kit provides the purification of total DNA. DNA binds specifically to the QIAamp silica-gel membrane while contaminants pass through. The main application of this method is to provide silica-based nucleic acid purification from blood samples.

### 4.4. Treatment

Colorectal surgery was the initial therapeutic approach for stages III or below. Treatment with 5-fluorouracil-based chemotherapy was used alone or in combination with an anti-vascular endothelial growth factor (VEGF) agent (Bevacizumab) or anti-epidermal growth factor receptor (EGFR) antibodies (Cetuximab or Panitumumab) on a two-week basis. Bevacizumab or monoclonal anti-EGFR antibodies were selected depending on RAS mutational status and primary tumor location. One month-long post-surgery VTE prophylaxis using low-molecular-weight heparin (LMWH) was implemented for all patients who underwent surgery.

### 4.5. Statistical Analysis

Statistical analysis was performed using SPSS software v26.0. Survival probabilities were estimated by the Kaplan–Meier method and the log-rank test was performed to see if there was a difference between the variable levels in terms of survival and VTE risk. We also performed Cox regression analysis to determine the factors affecting both survival and thrombosis development. Overall survival (OS) was calculated from the start of treatment until the date of death. Continuous variables that had a normal distribution were presented as mean and standard deviation (SD) or median and quartiles for those that did not have a normal distribution. The qualitative variables were presented as a number of cases (*n*) and percentages (%). Results with *p* < 0.05 were considered statistically significant.

## 5. Conclusions

To summarize, the results of our study show that the presence of activating KRAS mutation is an independent risk factor for VTE and, given the higher incidence in CRC patients, has the potential to become a relevant component in risk stratification models. Further studies are needed to characterize the link between KRAS mutation and VTE via heparanase production, increased TF expression, or both, and for the development of new risk stratification models that integrate genetic markers.

It’s important to note that while there is evidence suggesting an association between KRAS mutations and thrombosis in CRC, the relationship is complex and may be influenced by various factors, including the specific KRAS mutation type and the overall clinical characteristics of the patient. Therefore, further research is needed to fully elucidate the mechanisms and clinical implications of this association.

That being said, we believe that in clinical practice, healthcare providers should carefully assess the thrombotic risk in CRC patients, taking into account various factors, including genetic mutations like KRAS, to provide appropriate prophylactic or therapeutic interventions when necessary.

## Figures and Tables

**Table 1:** Baseline clinical characteristics of patients.

	KRAS-Mutated Patients (*n* = 43)	KRAS Wild-Type Patients (*n* = 87)
Age, years, mean (SD)	66.7 (9.5)	68.2 (12.1)
Gender, *n* (%)		
Male	18 (30%)	42 (70%)
Female	25 (35.71%)	45 (64.29%)
ECOG, *n* (%)		
0–1	31 (33.33%)	62 (66.66%)
≥2	12 (32.43%)	25 (67.56%)
Treatment, *n* (%)		
Bevacizumab	27 (84.37%)	5 (15.63%)
Other	16 (16.32%)	82 (83.68%)
TNM Stage, *n* (%)		
II	0 (0%)	7 (100%)
III	14 (26.41%)	39 (73.59%)
IV	29 (41.42%)	41 (58.57%)
VTE, *n* (%)		
DVT	13 (43.33%)	17 (56.66%)
PE	10 (66.66%)	5 (33.33%)
No VTE	20 (23.53%)	65 (76.47%)
Khorana score, *n* (%)		
<2	28 (25.69%)	81 (74.31%)
≥2	15 (71.42%)	6 (28.57%)

KRAS = Kirsten Rat Sarcoma Viral Oncogene Homologue; VTE = Venous thromboembolic events; ECOG = Eastern Cooperative Oncology Group.

**Table 2:** Patient survival rate stratified by VTE development.

VTE+			VTE−		
Cumulative Proportion Surviving at the Time			Cumulative Proportion Surviving at the Time		
Time (Months)	Estimate	Std. Error	Time (Months)	Estimate	Std. Error
14	0.964	0.025	12	0.987	0.013
18	0.945	0.031	13	0.944	0.027
19	0.906	0.040	24	0.907	0.037
23	0.867	0.047	30	0.855	0.050
24	0.788	0.057	36	0.826	0.056
26	0.767	0.059	40	0.757	0.069
27	0.744	0.062	45	0.673	0.083
28	0.699	0.066	48	0.631	0.088
36	0.652	0.069	65	0.541	0.113
38	0.627	0.071	68	0.451	0.125
40	0.602	0.072	96	0.225	0.171
45	0.552	0.074			
48	0.502	0.076			
55	0.443	0.077			
59	0.413	0.078			
60	0.325	0.076			
64	0.284	0.077			
84	0.213	0.084			
90	0.071	0.064			

Log-Rank *p* = 0.001; VTE = Venous thromboembolic events.

**Table 3:** Univariate and multivariate Cox regression analyses to identify predictors for increased risk of death in colorectal cancer patients.

Dependent Variable: Death			Univariate Analysis		Multivariate Analysis	
	Tested	Reference	HR (95% CI)	*p*-Value	HR (95% CI)	*p*-Value
Age	>65	<65	1.455 (0.97–3.11)	0.040	1.388 (0.76–2.34)	0.050
Sex	Male	Female	2.011 (1.12–4.11)	0.014	1.498 (0.94–2.43)	0.103
ECOG	ECOG 2–3	ECOG 0–1	1.757 (1.22–3.44)	0.009	1.329 (1.02–3.42)	0.010
KRAS	KRAS mt	KRAS wt	1.134 (0.84–2.88)	0.236	1.045 (0.96–2.53)	0.302
Stage	IV	II–III	1.721 (1.13–3.87)	0.040	1.688 (1.05–3.66)	0.090
Khorana Score	≥2	<2	3.718 (2.01–5.34)	0.001	3.131 (1.87–4.95)	0.020
VTE	VTE+	VTE−	1.952 (1.19–4.22)	0.026	1.475 (1.10–3.96)	0.077

KRAS = Kirsten Rat Sarcoma Viral Oncogene Homologue; VTE = Venous thromboembolic events; ECOG = Eastern Cooperative Oncology Group.

**Table 4:** Univariate logistic regression analysis to assess the predictive value of KRAS for VTE, DVT, and PE.

Independent Variable: KRAS		Non-Adjusted OR *p*-Value	Adjusted OR for Khorana Score (95% CI) *p*-Value	Adjusted OR for Bevacizumab Use (95% CI) *p*-Value	Adjusted OR for Stage (95% CI) *p*-Value
Dependent variable:	VTE	2.758 (1.18–7.23)	2.810 (1.23–7.54)	2.775 (1.34–7.66)	2.713 (1.11–7.45)
		*p* = 0.001	*p* = 0.004	*p* = 0.001	*p* = 0.04
	DVT	3.125 (1.72–7.66)	3.07 (1.62–6.89)	3.210 (1.77–6.22)	3.145 (1.54–6.49)
		*p* = 0.002	*p* = 0.003	*p* = 0.004	*p* = 0.03
	PE	1.752 (0.97–4.12)	1.650 (0.84–3.95)	1.832 (0.95–7.13)	1.778 (1.01–3.44)
		*p* = 0.045	*p* = 0.04	*p* = 0.001	*p* = 0.005

VTE—Venous thromboembolic events; DVT—Deep venous thrombosis; PE—Pulmonary embolism; OR—odd ratio; CI—confidence interval.

**Table 5:** VTE development stratified by KRAS mutational status.

KRAS—Wild Type			KRAS—Mutant		
Cumulative Proportion Surviving at the Time			Cumulative Proportion Surviving at the Time		
Time (Months)	Estimate	Std. Error	Time (Months)	Estimate	Std. Error
2	0.010	0.010	3	0.029	0.028
3	0.030	0.017	4	0.057	0.039
4	0.050	0.022	6	0.086	0.047
5	0.060	0.024	7	0.114	0.054
6	0.070	0.026	8	0.200	0.068
7	0.080	0.027	9	0.257	0.074
8	0.090	0.029	10	0.429	0.084
10	0.151	0.036	12	0.514	0.084
12	0.202	0.040	15	0.547	0.085
14	0.212	0.041	16	0.611	0.084
16	0.224	0.042	17	0.676	0.082
18	0.261	0.045	18	0.741	0.077
24	0.289	0.048	19	0.773	0.074
28	0.307	0.050	21	0.806	0.070
48	0.346	0.060	22	0.838	0.065
			29	0.892	0.062
			60	0.100	0.000

Log-Rank *p* < 0.001; KRAS = Kirsten Rat Sarcoma Viral Oncogene Homologue.

**Table 6:** Univariate and multivariate Cox regression analyses to identify predictors for increased risk of VTE in colorectal cancer patients.

Dependent Variable: VTE			Univariate Analysis		Multivariate Analysis	
	Tested	Reference	HR (95% CI)	*p*-Value	HR (95% CI)	*p*-Value
Age	>65	<65	1.209 (0.877–2.355)	0.340	1.109 (0.490–2.867)	0.490
Sex	Male	Female	1.800 (0.976–3.319)	0.050	1.698 (0.923–3.178)	0.036
ECOG	ECOG 2–3	ECOG 0–1	1.757 (0.803–2.433)	0.009	2.030 (1.152–3.579)	0.014
KRAS	KRAS mt	KRAS wt	3.252 (1.805–5.860)	0.001	2.538 (1.317–4.890)	0.005
Stage	IV	II–III	1.721 (0.963–3.125)	0.060	0.950 (0.421–2.148)	0.450
Khorana Score	≥2	<2	2.122 (1.233–3.166)	0.001	1.466 (0.976–3.288)	0.015
Treatment	Bevacizumab	Other	1.952 (1.101–2.985)	0.026	1.759 (0.977–4.125)	0.060

KRAS = Kirsten Rat Sarcoma Viral Oncogene Homologue; VTE = Venous thromboembolic events; ECOG = Eastern Cooperative Oncology Group.

## Data Availability

The data presented in this study are available on reasonable request from the corresponding authors.
